# Routine Primary Prophylaxis for Febrile Neutropenia with Biosimilar Granulocyte Colony-Stimulating Factor (Nivestim) or Pegfilgrastim Is Cost Effective in Non-Hodgkin Lymphoma Patients undergoing Curative-Intent R-CHOP Chemotherapy

**DOI:** 10.1371/journal.pone.0148901

**Published:** 2016-02-12

**Authors:** Xiao Jun Wang, Tiffany Tang, Mohamad Farid, Richard Quek, Miriam Tao, Soon Thye Lim, Hwee Lin Wee, Alexandre Chan

**Affiliations:** 1 Department of Pharmacy, National University of Singapore, Singapore, Singapore; 2 Department of Pharmacy, National Cancer Centre Singapore, Singapore, Singapore; 3 Division of Medical Oncology, National Cancer Centre Singapore, Singapore, Singapore; The University of York, UNITED KINGDOM

## Abstract

**Objective:**

This study aims to compare the cost-effectiveness of various strategies of myeloid growth factor prophylaxis for reducing the risk of febrile neutropenia (FN) in patients with non-Hodgkin lymphoma in Singapore who are undergoing R-CHOP chemotherapy with curative intent.

**Methods:**

A Markov model was created to compare seven prophylaxis strategies: 1) primary prophylaxis (PP) with nivestim (biosimilar filgrastim) throughout all cycles of chemotherapy; 2) PP with nivestim during the first two cycles of chemotherapy; 3) secondary prophylaxis (SP) with nivestim; 4) PP with pegfilgrastim throughout all cycles of chemotherapy; 5) PP with pegfilgrastim during the first two cycles of chemotherapy; 6) SP with pegfilgrastim; and 7) no prophylaxis (NP). The perspective of a hospital was taken and cost-effectiveness was expressed as the cost per episode of FN avoided over six cycles of chemotherapy. A probabilistic sensitivity analysis was conducted.

**Results:**

Strategies 3, 6, and 7 were dominated in the base case analysis by strategy 5. The costs associated with strategies 2, 5, 1, and 4 were US$3,813, US$4,056, US$4,545, and US$5,331, respectively. The incremental cost-effectiveness ratios for strategy 5 vs. strategy 2, strategy 1 vs. strategy 5, and strategy 4 vs. strategy 1 were US$13,532, US$22,565, and US$30,452, respectively, per episode of FN avoided. Strategy 2 has the highest probability to be cost-effective (ranged from 48% to 60%) when the willingness to pay (WTP) threshold is lower than US$10,000 per FN episode prevented.

**Conclusion:**

In Singapore, routine PP with granulocyte colony-stimulating factor (nivestim or pegfilgrastim) is cost-effective for reducing the risk of FN in patients receiving R-CHOP.

## Introduction

Febrile neutropenia (FN) is a major complication of myelosuppressive chemotherapy. Management of this complication is often associated with a high economic burden during cancer care [1]. Diffuse large B-cell lymphoma (DLBCL) is the most common subtype of non-Hodgkin lymphoma (NHL). The first-line treatment for DLBCL includes combination chemotherapy with cyclophosphamide, doxorubicin, vincristine, prednisone, and rituximab (R-CHOP) [2]. The current guidelines suggest that patients who are undergoing R-CHOP chemotherapy are at intermediate risk (10% to 20%) for FN and it is recommended that the prophylactic use of granulocyte colony-stimulating factor (G-CSF) should be considered among patients with risk factors for FN, such as old age, previous chemotherapy or radiotherapy, and poor performance status [3, 4].

In Singapore, two types of G-CSF are commercially available, filgrastim and pegfilgrastim (a second-generation filgrastim with a sustained duration of action). Because the patent for filgrastim recently expired, a number of biosimilars of filgrastim are currently under evaluation and development. Nivestim, a biosimilar filgrastim, was recently approved for clinical use in Singapore. In a Phase 3 clinical trial, the efficacy and safety of nivestim were demonstrated to be similar to those of filgrastim [5]. Because the price of biosimilar filgrastim (nivestim) has the potential to be lower than that of its reference product (filgrastim), there is an opportunity for nivestim to improve the cost-effectiveness of the prophylaxis strategies for FN.

Although several cost-effectiveness analyses of the prophylactic use of G-CSF among lymphoma patients who are undergoing CHOP-based chemotherapy have been published in the past few years [6–9], the results have been varied (Table 1). Recently, one study from the European G5 countries showed that the prophylactic use of biosimilar filgrastim was more cost-efficient for the reduction of FN under all possible prophylaxis strategies evaluated than filgrastim and pegfilgrastim [10]. However, to the best of our knowledge, publications regarding the cost-effectiveness of FN prophylaxis with biosimilar G-CSF among lymphoma patients who are undergoing R-CHOP chemotherapy are limited. Therefore, the primary objective of this study was to compare the cost-effectiveness of primary and secondary prophylaxis with biosimilar filgrastim (nivestim), pegfilgrastim, or no prophylaxis to reduce the risk of FN in patients with NHL who were undergoing R-CHOP chemotherapy with curative intent, through the perspective of a Singaporean hospital.

**Table 1:** A rapid review of cost-effectiveness analyses of G-CSF prophylaxis among lymphoma patients undergoing CHOP-based chemotherapy.

Study	Hill G et al. [6]	Lathia N et al. [7]	Chan KK et al. [8]	Lyman G et al. [9]
Year	2014	2013	2012	2009
Location	United States	Canada	Canada	United States
Perspective	Government payer	Healthcare systems	Government payer	Health insurers
Design	Decision tree + Markov cohort	Markov cohort	Markov cohort	Decision tree + Markov cohort
Population	66-year-old NHL patients (modeled)	64-year-old DLBCL patients (modeled)	Newly diagnosed DLBCL patients (modeled)	Intermediate / high-grade NHL (modeled)
Comparators	1) PP with pegfilgrastim 2) SP with pegfilgrastim	1) NP 2) PP with 10 days of filgrastim 3) PP with pegfilgrastim	1) PP with 10 days of filgrastim 2) SP with 10 days of filgrastim	1) PP with pegfilgrastim 2) PP with 6 days of filgrastim
Time horizon	Lifetime	6 cycles of chemotherapy (18 weeks)	8 cycles of chemotherapy	Lifetime
Cost	Direct medical cost (2012 USD)	Direct medical cost (2012 CAD)	Direct medical cost (2010 CAD)	Direct medical cost (2006 USD)
Outcomes	LYS; QALY gained; FN event avoided	QALY gained	QALY gained	LYS; QALY gained; FN event avoided
Main Results	ICERs for #1 vs. #2 were $15,000 per FN event avoided, $33,000 per QALY gained, and $28,900 per LYS; PP with pegfilgrastim was cost-effective vs. SP with pegfilgrastim at a WTP threshold of $50,000 per QALY	ICERs for #2 vs. #1 and #3 vs. #2 were CAD $5,796,000 per QALY and CAD $2,611,000 per QALY, respectively; Neither PP with pegfilgrastim nor PP with filgrastim were cost-effective vs. NP	ICER for #1 vs. #2 was $700,500 per QALY gained; PP with filgrastim was not cost-effective vs. SP with filgrastim at a WTP threshold of $100,000/QALY	ICERs for strategy #1 vs. #2 were $2,167/FN episode avoided, $5,532 per LYS, and $6,190 per QALY gained; PP with pegfilgrastim was cost-effective vs. PP with filgrastim

**NP =** no prophylaxis; **PP =** primary prophylaxis; **SP =** secondary prophylaxis; **pts =** patients; **USD =** US dollars; **CAD =** Canada dollars; **QALY =** quality-adjusted life year; **LYS =** life year saved; **ICER =** incremental cost-effectiveness ratio; **WTP =** willingness-to-pay

## Methods

### Model overview

A Markov model was constructed with TreeAge Pro 2013 (TreeAge Software, Inc, MA) to compare seven prophylaxis strategies for FN: 1) primary prophylaxis (PP) with nivestim through all cycles of chemotherapy; 2) PP with nivestim during the first two cycles of chemotherapy; 3) secondary prophylaxis (SP) with nivestim; 4) PP with pegfilgrastim through all cycles of chemotherapy; 5) PP with pegfilgrastim during the first two cycles of chemotherapy; 6) SP with pegfilgrastim; and 7) no prophylaxis (NP). Primary prophylaxis is defined as the routine administration of G-CSF with each cycle of chemotherapy, regardless whether a patient had previously experienced an episode of FN. Secondary prophylaxis is defined as the initation of G-CSF in subsequent cycles of chemotherapy after a patient experienced a FN episode. For both SP strategies (strategies 3 and 6) and those that included PP at the first two cycles of chemotherapy (strategies 2 and 5), secondary G-CSF prophylaxis was initiated and continued during the subsequent cycles once the patient experienced an episode of FN.

The model target population was a hypothetical cohort of patients with NHL (mean age, 55 years) with R-CHOP as a curative treatment. The time horizon of this model was 18 weeks, which was the period of six chemotherapy cycles. The Markov model was used with a cycle length of 1 week (7 days).

### Model structure

**Fig 1:**
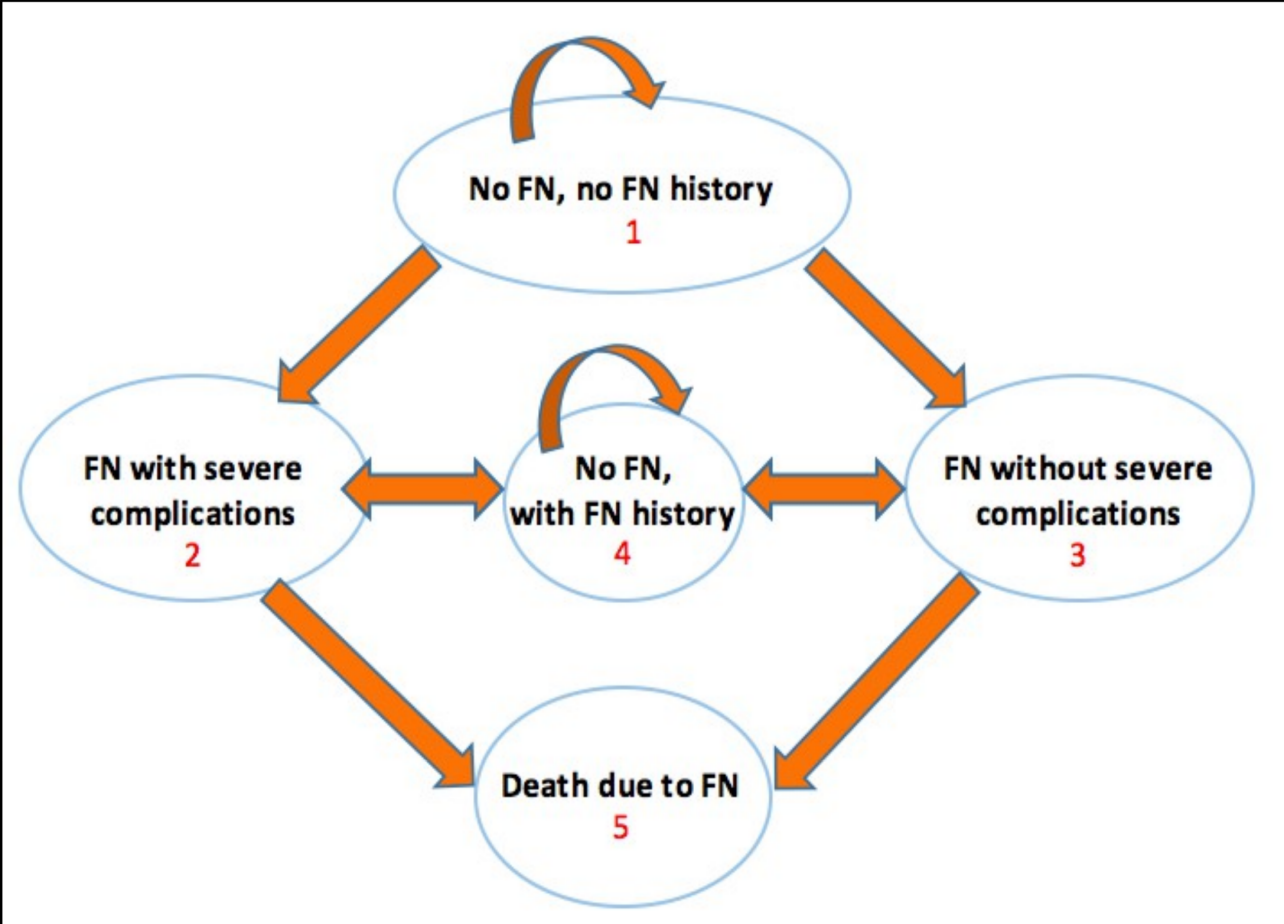
Structure of Markov state transition model.

This Markov model included five health states: 1) no FN or history of FN; 2) FN with severe complications; 3) FN without complications; 4) no FN, but a history of FN; and 5) death of FN (Fig 1). Death of other causes during chemotherapy was not considered.

**Fig 2:**
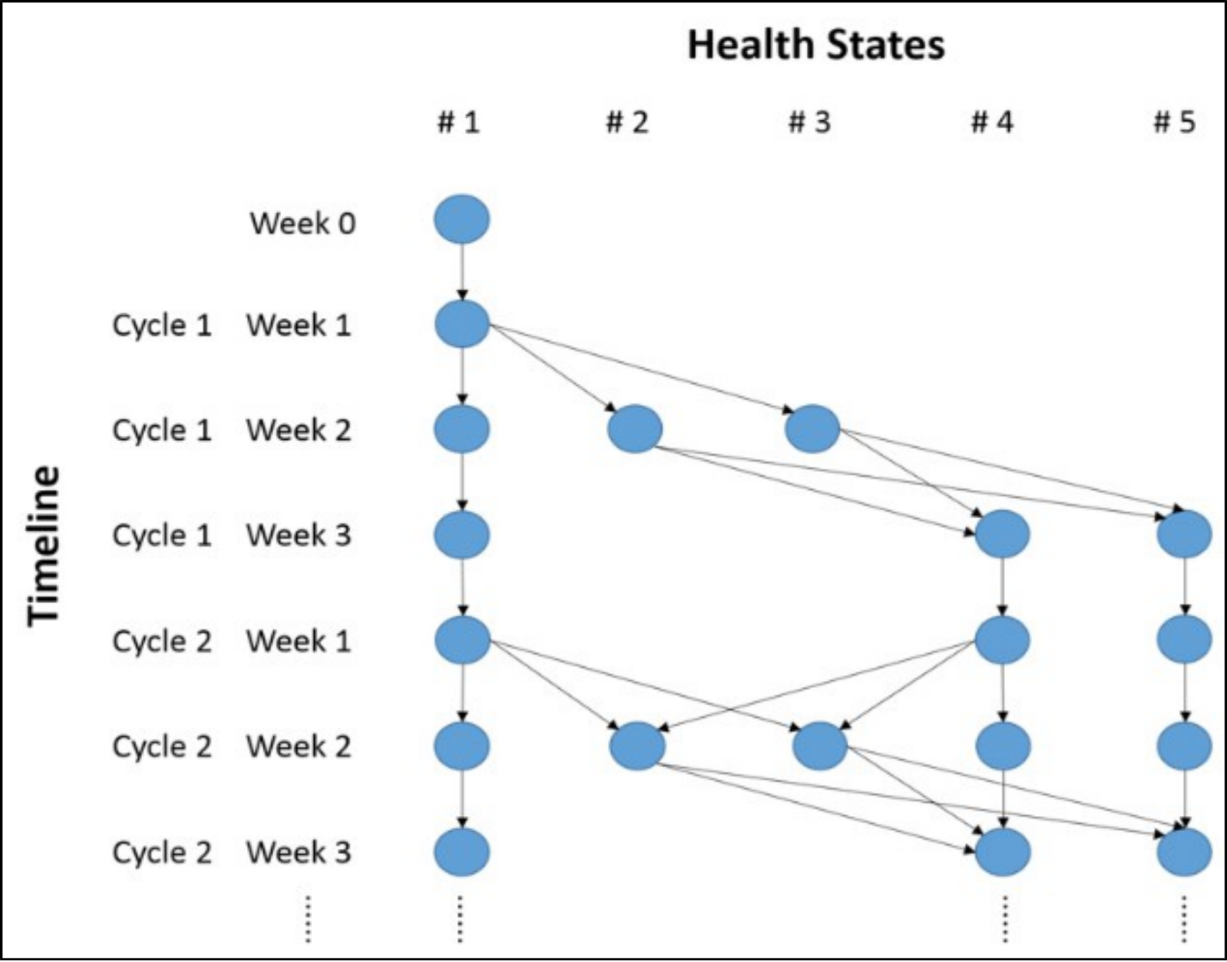
Markov trace of the cohort simulation for the first two cycles of chemotherapy.

In this model, all patients began in health state 1 (no FN or history of FN). Health states 2 and 3 were temporary states that individuals had to exit after 1 week. To integrate the memory of FN history into the Markov model, health state 4 was added. Health state 5 was set as the absorbing state. The transitions from state 1 to states 2 and 3, and from state 4 to states 2 and 3 only occurred in week 2 of each chemotherapy cycle (Fig 2). The face validity of this model has been discussed and confirmed with local experts and clinicians. This study was approved by the SingHealth Institutional Review Board. Informed consent was waived, and patient records/information was anonymized and de-identified prior to analysis.

### Model inputs

#### Cost

The cost data on nivestim and pegfilgrastim were obtained from National Cancer Center Singapore (NCCS). The cost of medication administration was not included because the patients self-inject G-CSF based on local practice (Table 2).

**Table 2:** Model inputs.

Parameters	Value	Reference	PSA distribution
**Costs, 2013 USD**			
Pegfilgrastim 6 mg (per injection)	532 USD	NCCS	N/A
Nivestim 300 μg (7 days of injections)	352 USD	NCCS, [17]	N/A
Hospitalization cost for FN with complications	22,135 USD	NCCS, [11]	Gamma (α = 29.23, λ = 0.0013)
Hospitalization cost for FN without complications	9,588 USD	NCCS, [11]	Gamma (α = 98.72, λ = 0.010)
**Utilities**			
NHL with chemotherapy (without FN)	0.80	NCCS, [15]	Beta (α = 92.70, ß = 23.17)
FN without complications	0.77	NCCS, [15]	Beta (α = 61.33, ß = 18.42)
FN with severe complications	0.43	NCCS, [15]	Beta (α = 4.43, ß = 5.78)
**Probabilities and efficacy**			
Relative risk of FN (versus no G-CSF); pegfilgrastim	0.55	[17, 19]	Log-normal (mean = -0.60, SD = 0.22)
Relative risk of FN (versus no G-CSF); nivestim	0.62	[18, 19]	Log-normal (mean = -0.48, SD = 0.13)
Relative risk of FN; with FN history (versus no FN history)	2.29	NCCS, [17]	Log-normal (mean = 0.83, SD = 0.40)
FN risk in cycle 1; no prophylaxis	0.17	[19]	Beta (α = 84, ß = 410)
FN risk in cycles 2 to 6; no prophylaxis	0.03	[19]	Beta (α = 61, ß = 349)
Risk of severe complications in cases of FN	0.14	NCCS, [11]	Beta (α = 13, ß = 83)
FN with complications case fatality rate	0.025	[21]	Beta (α = 4, ß = 154)
FN without complications case fatality rate	0.0063	[21]	Beta (α = 1, ß = 157)

**USD =** US dollars; **N/A =** not available; **NCCS =** National Cancer Centre Singapore; **PSA** = Probabilistic sensitivity analysis; **G-CSF =** granulocyte colony-stimulating factor; **FN =** Febrile neutropenia; **SD =** Standard deviation; **NHL** = non Hodgkin’s lymphoma

Although episodes of FN may lead to a dose reduction or a delay in chemotherapy that may affect the patient’s treatment cost, for this study, the variation on treatment costs was limited because the simulated cohort of patients were undergoing curative-intent chemotherapy. It was therefore assumed that the costs of chemotherapy were the same across all arms of the model, so these costs were not included in our analysis.

The costs of hospitalization for FN among lymphoma patients with and without serious complications were obtained through an observational study conducted at the NCCS [11]. A previous study in Singapore reported that more than 95% of cases of FN were managed in an inpatient setting [12]. To simplify the model, all FN events in this study were assumed to require hospitalization.

All costs were first adjusted to 2013 Singapore dollars by the Singapore consumer price index (health care component) [13]. The cost data were then converted to US dollars using the 2013 purchasing power parity conversion rate of 1 USD = 0.88 SGD [14], obtained from the World Bank.

#### Utilities

Utility estimates were generated from a local study in Singapore [15]. In that study, there are a total of 67 hospitalized patients with lymphoma who were undergoing chemotherapy. Twenty-eight out of 67 patients (41.8%) developed FN within the past 7 days from the interview date, among which 7 patients (25%) experienced severe complications. The EuroQol 5-Dimensions questionnaire (EQ-5D) was administered to each patient, and the average utility index score was calculated to estimate the utility value for each health state. EQ-5D utility index scores were calculated using a Japanese value set, which offers the closest approximation to Singapore’s population [16].

#### Probabilities and efficacy

Table 2 shows the efficacy of pegfilgrastim in the prevention of FN in patients with NHL estimated with data from a local retrospective study [17]. We assumed that nivestim had an efficacy equivalent to that of filgrastim [5] and estimated its efficacy on the basis of a randomized trial in lymphoma patients [18]. Nivestim was assumed to be given for 7 days per cycle of chemotherapy [17].

The relative risk (RR) of FN with and without a history of FN within each chemotherapy cycle was based on an observational study that was conducted by the NCCS [17]. The risk of severe complications in cases of FN was estimated in a study conducted in Singapore [11].

In addition, the risks of the development of FN without PP with G-CSF were obtained from Lyman et al. [19]. It was assumed that R-CHOP had a risk of FN equivalent to that of the CHOP regimen [20]. The risks of the development of FN in cycles 2 to 6 were assumed to be equivalent [19].

The case-fatality rates for FN with and without severe complications in patients with NHL were based on a prospective observational study [21] from Singapore.

### Model analyses

The model outputs included the total costs, the number of FN episodes prevented, and the quality-adjusted life years (QALYs) gained for each of the seven prophylaxis strategies. The primary outcome of this economic evaluation was the incremental cost per episode of FN prevented. If a strategy was more costly and did not provide any additional benefit (i.e., both more costly and less effective), it was considered to be “dominated.”

A probabilistic sensitivity analysis was performed and a beta distribution was used to reflect the uncertainty in the risk. The cost parameters were assumed to follow a gamma distribution. A log-normal distribution was used for the RR. The parameters of these distributions were derived from published literature when available [11, 17–19, 21]. The Monte Carlo simulation was conducted for 10,000 iterations for each comparison. The cost-effectiveness acceptability curves were generated on the basis of the results of the probabilistic sensitivity analysis.

A scenario analysis was performed. The cost per QALY gained was chosen as the outcome for each individual prophylactic strategies. In addition, a threshold analysis was conducted, of which the cost of nivestim was varied downward until the PP with nivestim (all cycles) became the most cost-effective prophylaxis strategy. A cost-effectiveness threshold of US$100,000 per QALY gained was employed.

## Results

### Base-case analysis

Table 3 shows the total and incremental health outcomes and costs associated with all seven FN management strategies (cost per FN episode prevented as the main outcome). NP, SP with nivestim, and SP with pegfilgrastim were dominated by PP with pegfilgrastim (cycles 1 and 2). The costs associated with PP with nivestim (cycles 1 and 2), PP with pegfilgrastim (cycles 1 and 2), PP with nivestim (all cycles), and PP with pegfilgrastim (all cycles) were US$3,813, US$4,056, US$4,545, and US$5,331, respectively. The number of FN episodes per patient throughout all six cycles of chemotherapy were 0.25, 0.24, 0.21, and 0.19, respectively.

**Table 3:** Results of the costs and effectiveness of the prophylaxis strategies (cost per FN episode prevented).

Strategy	Cost, 2013 USD	Episode of FN per patient ^@^	Incremental cost, 2013 USD	Incremental FN episode prevented	ICER, 2013 USD
PP with nivestim (cycles 1 & 2)	3,813	0.25	reference	reference	reference
PP with pegfilgrastim (cycles 1 & 2)	4,056	0.24	243	0.02	13,532
PP with nivestim (all cycles)	4,545	0.21	489	0.02	22,565
PP with pegfilgrastim (all cycles)	5,331	0.19	786	0.03	30,452
No prophylaxis *	4,101	0.36	—	—	Dominated
SP with nivestim *	4,162	0.33	—	—	Dominated
SP with pegfilgrastim *	4,297	0.33	—	—	Dominated

^@^ Throughout all six cycles of chemotherapy

* Dominated by PP with pegfilgrastim (cycles 1 and 2)

**PP =** Primary prophylaxis; **SP =** Secondary prophylaxis; **FN =** Febrile neutropenia; **USD =** US dollars; **ICER** = Incremental cost-effectiveness ratio

The incremental cost-effectiveness ratio (ICER) for PP with pegfilgrastim (cycles 1 and 2) compared with PP with nivestim (cycles 1 and 2) was US$13,532 per episode of FN prevented; for PP with nivestim (all cycles) compared with PP with pegfilgrastim (cycles 1 and 2), the ICER was US$22,565 per episode of FN prevented; and for PP with pegfilgrastim (all cycles) compared with PP with nivestim (all cycles), the ICER was US$30,452 per episode of FN prevented (Table 3).

### Probabilistic sensitivity analysis

**Fig 3:**
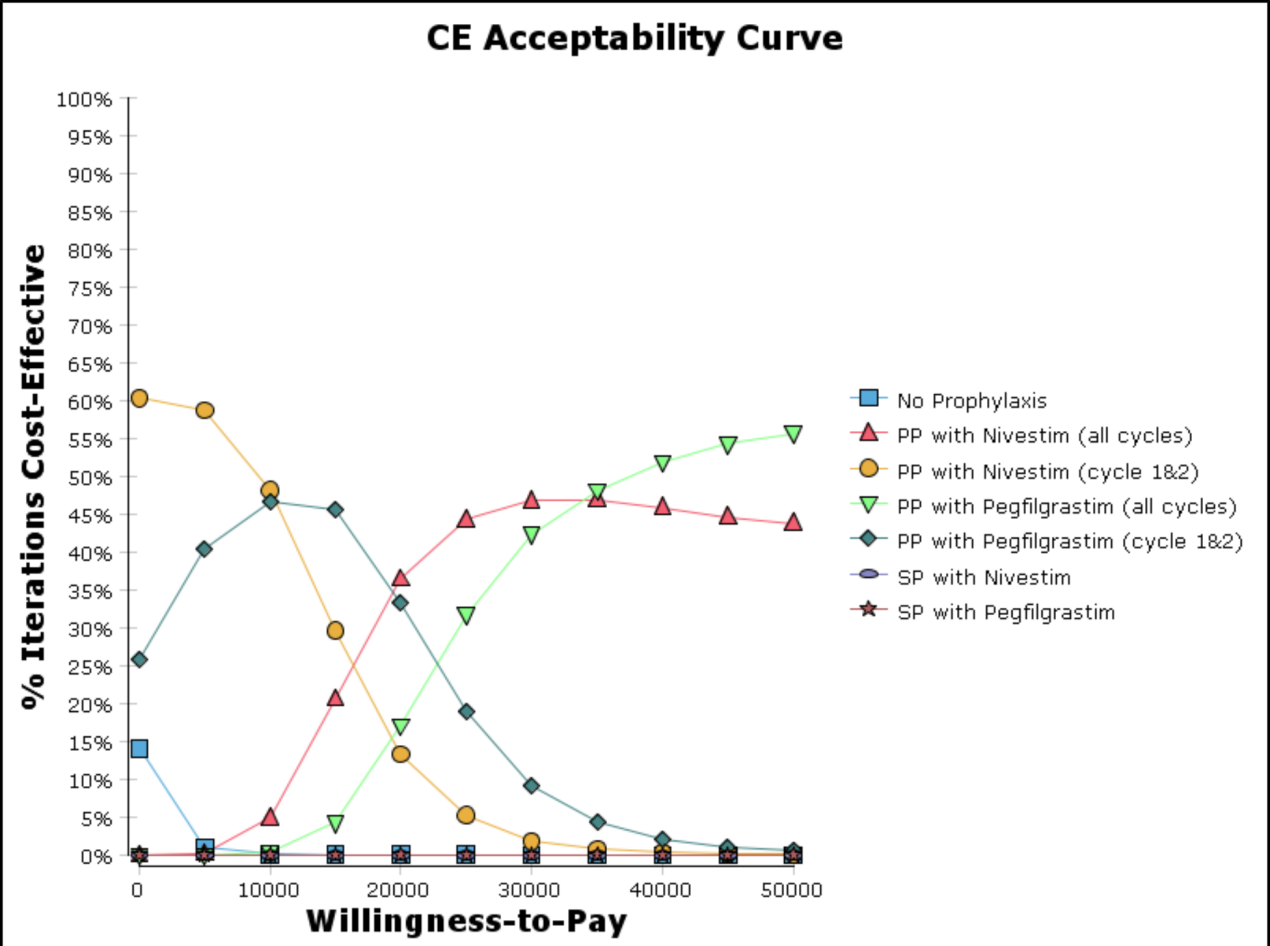
Cost-effective acceptability curve (cost per FN episode prevented).

The cost-effectiveness acceptability curve from the probabilistic sensitivity analysis (Fig 3) revealed that PP with nivestim (cycles 1 and 2) has the highest probability to be cost-effective (ranged from 48% to 60%) when the willingness to pay (WTP) threshold is lower than US$10,000 per FN episode prevented. In contrast, if the WTP threshold is higher than US$20,000 per FN episode prevented, PP with G-CSF (nivestim or pegfilgrastim) for all cycles would become the prophylaxis strategy with the highest probability to be cost-effective.

### Scenario analysis

Table 4 shows the total and incremental health outcomes and costs associated with all seven FN management strategies (cost per QALY gained as the main outcome). NP, SP with nivestim, and SP with pegfilgrastim were dominated by PP with pegfilgrastim (cycles 1 and 2). PP with nivestim (all cycles) was extended dominated by the mixed strategy of PP with pegfilgrastim (cycles 1 and 2) and PP with pegfilgrastim (all cycles). When the cost of nivestim (7 days of injections) was lower than US$137, PP with nivestim throughout all cycles of chemotherapy would become cost-effective at a threshold of US$100,000 per QALY gained.

**Table 4:** Results of the costs and effectiveness of the prophylaxis strategies (cost per QALY gained).

Strategy	Cost, 2013 USD	QALYs ^@^	Incremental cost, 2013 USD	Incremental QALYs gained	ICER, 2013 USD
PP with nivestim (cycles 1 & 2)	3,813	0.2754	reference	reference	reference
PP with pegfilgrastim (cycles 1 & 2)	4,056	0.2755	243	0.0001	4,058,623
PP with pegfilgrastim (all cycles)	5,331	0.2756	1,275	0.0001	11,928,289
PP with nivestim (all cycles) ^	4,545	0.2755	—	—	Dominated
No prophylaxis *	4,101	0.2751	—	—	Dominated
SP with nivestim *	4,162	0.2733	—	—	Dominated
SP with pegfilgrastim *	4,297	0.2752	—	—	Dominated

^@^ Throughout all six cycles of chemotherapy

* Dominated by PP with pegfilgrastim (cycles 1 and 2)

^ Extended dominated by a mixed strategy of PP with pegfilgrastim (cycle 1&2) and PP with pegfilgrastim (all cycles)

**PP =** Primary prophylaxis; **SP =** Secondary prophylaxis; **FN =** Febrile neutropenia; **QALYs** = quality-adjusted life years; **USD =** US dollars; **ICER** = Incremental cost-effectiveness ratio

## Discussion

In our study, we found that NP, SP with nivestim, and SP with pegfilgrastim were dominated by PP with pegfilgrastim (cycles 1 and 2) in the base case analysis. This advocates for a routine PP strategy to prevent FN among all patients with NHL who undergo R-CHOP chemotherapy. We recently published a study of the economic burden of FN among patients in Singapore with solid tumors and lymphoma. The inpatient management cost of FN was US$4193 per episode, and the management cost of FN in lymphoma cases (US$6560) was the highest among the cancer types [11]. Hence, this emphasizes the importance to routinely PP patients with G-CSF (nivestim or pegfilgrastim) who are undergoing R-CHOP in Singapore in order to prevent the occurrence of FN, as this strategy is cost effective and it reduces patients’ risks of FN risks for hospitalization and infectious-related complications.

Considering the short time horizon (18 weeks) in our proposed model, it is not suitable to use QALYs as the primary outcome for this study, because the benefit of reducing the FN-related mortality rate using G-CSF cannot be fully captured within a relative short time horizon (during the chemotherapy cycles). Therefore, we have chosen the ICER involving natural health units (episodes of FN prevented) as the main outcome for this study. However, we have also conducted our analysis using the cost per QALY gained approach, in order to ensure our study findings are comparable to other cost-effectiveness studies [6–9]. It should be noticed that when the cost per QALY gained approach was used, multiple strategies (NP, SP with nivestim, and SP with pegfilgrastim) are still dominated by PP with pegfilgrastim (cycles 1 and 2) in the base case analysis. This demonstrates robustness of our main finding that a routine PP strategy to prevent FN should be advocated among all patients with NHL receiving R-CHOP chemotherapy.

A recent US study evaluated the cost-effectiveness of G-CSF prophylaxis among patients with NHL who were undergoing CHOP-based chemotherapy [6]. In that study, a time horizon of life was used and the cost-effectiveness was assessed by the ICER involving QALYs. PP with G-CSF was found to be cost-effective when compared with SP with G-CSF at a WTP threshold of US$50,000 per QALY. This is consistent with the findings in our study. A Canadian study [7] assessed the cost-effectiveness of PP with G-CSF among patients with DLBCL who underwent R-CHOP during six cycles of chemotherapy. It was reported that PP with G-CSF was not cost-effective when compared with NP at a WTP threshold of US$50,000 per QALY. The main reason why this study presents different results from the current study [6] could be due to the relatively short time horizon they have chosen and they have included the NP strategy as the reference (assuming NP strategy is the standard care in their settings). We believe that the assessment of the cost-effectiveness of G-CSF prophylaxis within a shorter time horizon (during the chemotherapy cycles) is more appropriate because FN is an acute illness and there is little evidence to support the long-term benefits of G-CSF prophylaxis. However, it was identified in our study that the NP strategy was dominated by the PP with G-CSF (nivestim or pegfilgrastim) for the first two cycles of chemotherapy. To our knowledge, this is the first study that has evaluated the value of PP with G-CSF during the first two cycles of chemotherapy among lymphoma patients. One previous study [22] concluded that G-CSF prophylaxis for all cycles of chemotherapy is more effective in patients with breast cancer, but associated with higher cost, when compared to the administration of prophylaxis solely for the first two cycles of chemotherapy. However, the cost-effectiveness of PP with G-CSF limited to the first two cycles has not been determined. The results in our study demonstrated that this strategy is cost-effective when compared to the PP with G-CSF for all cycles as this strategy generates a sizable cost-saving with a slight loss on the health benefits that may not be clinically significant. However, further studies are required to refine and optimize a dosing regimen that requires less G-CSF.

We identified that when the cost of nivestim (7 days of injections) was lower than US$137, PP with nivestim throughout all cycles of chemotherapy was cost-effective and should be routinely recommended as PP. This result implies that as the cost of biosimilar G-CSF continues to decrease, the use of G-CSF will be encouraged in such a way that patients’ access to the treatment will increase. It was recently reported that the availability of a lower-cost biosimilar G-CSF was associated with a 10% to 20% increase in the use of G-CSF in Europe [23]. This had a major effect on clinical practice, as many clinicians are moving away from SP to more routine PP [24]. However, the adoption of a biosimilar in clinical practice should not be driven only by monetary concerns but also by its safety and efficacy [24]. Two prospective observational studies of nivestim (biosimilar filgrastim) are currently underway [25]. It is expected that the long-term safety and efficacy data provided by these two studies will promote our understanding of the performance of nivestim in clinical practice. An additional cost-effectiveness analysis may be necessary in the future to compare the cost-effectiveness of other biosimilar filgrastim with its reference product (filgrastim).

Our study has several limitations. First, the efficacy of nivestim was assumed to be equivalent to that of filgrastim. Although it is safe for us to assume that their efficacies are similar, this uncertainty may have affected our results. Second, we presented all-cause mortality in our model, as we were unable to segregate the mortality of other causes during chemotherapy. This could potentially lead to an overestimation on both costs and effectiveness. However, given the short time horizon in this model (18 weeks) and most mortality during this period are related to FN, exclusion of this health state should not lead to significant impact.

In conclusion, routine PP with G-CSF (nivestim or pegfilgrastim) is cost-effective in Singapore for reducing the risk of FN in patients receiving R-CHOP than other strategies including secondary prophylaxis or not offering prophylaxis.

## Supporting Information

S1 Fig Cost-effective acceptability curve (cost per QALY gained). (DOCX)
