# Supplementary figures and images for: Routine Primary Prophylaxis for Febrile Neutropenia with Biosimilar Granulocyte Colony-Stimulating Factor (Nivestim) or Pegfilgrastim Is Cost Effective in Non-Hodgkin Lymphoma Patients undergoing Curative-Intent R-CHOP Chemotherapy
Source: PLoS One. 2016 Feb 12;11(2):e0148901. doi: 10.1371/journal.pone.0148901 (PMC4752449; doi:10.1371/journal.pone.0148901)

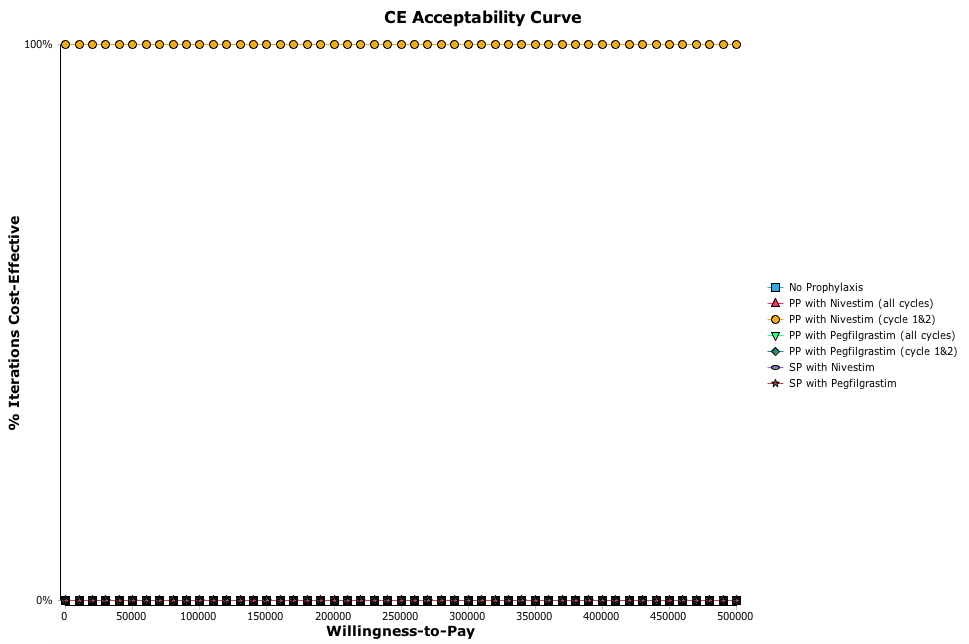


**S1 Fig. Cost-effective acceptability curve (cost per QALY gained)**

Supplement: S1 Fig — (DOCX) [file pone.0148901.s001.docx]
